# Risk factors for lower extremity amputation in patients with diabetic foot ulcers: A meta-analysis

**DOI:** 10.1371/journal.pone.0239236

**Published:** 2020-09-16

**Authors:** Chunmei Lin, Jinhao Liu, Hu Sun

**Affiliations:** 1 Department of Endocrinology and Metabolism, Fujian Medical University Xiamen Humanity Hospital, Xiamen, Fujian Province, People’s Republic of China; 2 Department of Vascular Surgery, Zhongshan Hospital Affiliated to Xiamen University, Xiamen, Fujian Province, People’s Republic of China; Suez Canal University Faculty of Medicine, EGYPT

## Abstract

**Purpose:**

A considerable number of diabetic foot ulcer (DFU) patients require amputation every year, which worsens their quality of life, aggravates the social burden, and shortens their life expectancy. Considering these negative effects, it is important to explore the relative risk factors affecting amputation in DFU patients.

**Methods:**

The PubMed, SCIE and Embase databases were comprehensively searched for prospective or retrospective studies published before October 31, 2019. All English language studies involving DFU patients were included, and RevMan 5.3 software was used to analyse the data.

**Results:**

This meta-analysis includes 21 studies involving 6505 participants, including 2006 patients who required a lower limb amputation. The following variables were associated with an increased risk of amputation: male sex (odds ratios (OR) = 1.30, 95% confidence interval (CI) = 1.16~1.46, P<0.00001), smoking history (OR = 1.19, 95% CI = 1.04~1.35, P = 0.009), a history of foot ulcers (OR = 2.48, 95% CI = 2.00~3.07, P<0.00001), osteomyelitis (OR = 3.70, 95% CI = 3.02~4.53, P<0.00001), gangrene (OR = 10.90, 95% CI = 5.73~20.8, P<0.00001), a lower body mass index (mean difference IV (MD) = -0.88, 95% CI = -1.30~-0.47, P<0.0001), and a higher white blood cell count (MD = 2.42, 95% CI = 2.02~2.82, P<0.00001). However, age (MD = 1.24, 95% CI = -0.45~2.93, P = 0.15), type of diabetes (OR = 0.96, 95% CI = 0.61~1.52, P = 0.86), hypertension (OR = 1.19, 95% CI = 0.96~1.47, P = 0.12), and HbA1c level (MD = 0.02, 95% CI = -0.28~0.33, P = 0.87) were not associated with amputation in patients with DFU.

**Conclusions:**

Our meta-analysis identified several risk factors for amputation in DFU patients, including the male sex, a smoking history, a history of foot ulcers, osteomyelitis, gangrene, a lower body mass index, and a higher white blood cell count. Once gangrene occurs, the risk of amputation rapidly increases.

## Introduction

Diabetes mellitus, which is among the most common endocrine diseases, severely deteriorates patients’ quality of life; even worse, this condition shortens their life expectancy. More than 415 million people worldwide suffer from diabetes. More seriously, the prevalence of diabetes is still rising, and it is expected that the number of people affected will surge to 640 million by 2040 [1]. Currently, up to 1/4 of diabetic patients can develop foot ulcers, and at least one-quarter of these ulcers do not heal, placing such patients at risk of amputation [2].

Diabetic foot ulcer (DFU) is the leading cause of hospitalization in diabetic patients and among the most common, severe and costly complications of diabetes mellitus, resulting in major medical, financial, and social consequences for the patients, their families and society in general [3]. Approximately 40% to 60% of nontraumatic lower limb amputations worldwide are caused by diabetic complications, and 80% of these amputations follow diabetic foot ulcer [4]. Previous studies have shown that amputations (including major and minor amputations) caused by diabetes have a high mortality rate with a 5-year survival rate of 41% to 48% [5, 6]. Even in patients with minor amputations, the 5-year survival rate is only 59% [5].

Knowledge of the risk factors for amputation can be helpful for patients newly diagnosed with DFU. Considering the above reasons, it is necessary to be able to identify the relative risk factors for lower extremity amputation (LEA) in patients with DFU that can be modified to avoid or delay the severe consequences. To address these issues, we performed a meta-analysis to assess the risk factors associated with LEA in DFU patients.

## Methods

This meta-analysis was based on the preferred reporting items for systematic reviews and meta-analyses (PRISMA) statement [7].

### Search strategy

The PubMed, Embase and Web of Science databases were used to conduct a systematic literature search for studies published through October 31, 2019. The following terms were used for DFU: “Diabetic Feet” OR “Diabetic Foot” OR “Foot Ulcer, Diabetic”. The keywords used for the risk factors included “Predictive factors” OR “Predictive factor” OR “Risk Factors” OR “Risk Factor” OR “Population at Risk” OR “Predictors”. The terms associated with amputation included “Amputation” OR “Limb loss”. The three authors carried out an independent selection process and resolved their differences through discussion.

### Inclusion and exclusion criteria

In this meta-analysis, the included studies had to meet the following criteria: (i) the articles were prospective or retrospective publications based on original data; (ii) the articles were published in the English language; (iii) all patients were diagnosed with DFU regardless of the diabetes type; (iv) all patients were diagnosed with DFU with or without a history of amputation or ulcers; and (v) the demographics and clinical characteristics of the DFU patients were available for the data extraction. Studies meeting the following exclusion criteria were eliminated from this meta-analysis: (i) reviews, letters to the editor, commentaries and editorials, irretrievable articles, animal studies and other studies from which patient data could not be extracted; (ii) studies in which the full text was not written in the English language; and (iii) simple diabetic foot patients without ulcers or simple diabetic foot infection patients. Two independent authors screened all titles and abstracts to determine eligibility. The full texts were browsed when eligibility could not be determined by the abstracts, and any discrepancies were resolved through discussion. All studies identified by the search strategy were exported to Endnote X8 software.

### Data extraction and quality assessment

Two authors extracted the relevant data from the included articles into a structured table. The first author, year of publication, country and region, research design, number of cases, incidence, potential risk factors and corresponding data were recorded independently. Two researchers evaluated the quality of the studies independently using the Newcastle–Ottawa Scale (NOS). The NOS was used to assess the risk of bias based on the following three major components: (i) “group selection” (up to 4 points); (ii) “comparability” (up to 2 points); and (iii) “assessment of outcome or exposure” (up to 3 points). The total NOS score of each study ranged from 0 to 9 [8], and the studies were considered high-quality studies if they scored equal to or greater than 5 [9].

### Statistical analysis

The statistical analysis of the data was performed using RevMan 5.3 software. The results are presented as the mean difference IV (MD) or odds ratios (ORs) with a 95% confidence interval (CI), and a P-value<0.05 was considered statistically significant unless otherwise specified. In addition, heterogeneity was quantified using the Q test and I^2^ statistics. When the heterogeneity test indicated no significant difference (P>0.1 and I^2^<50%), a fixed-effects model was applied; otherwise, a random-effects model was used. Begg’s funnel plot test was used to assess possible publication bias. A trial sequential analysis (TSA) was used to determine whether the sample size is sufficient to obtain significant results.

## Results

**Fig 1:**
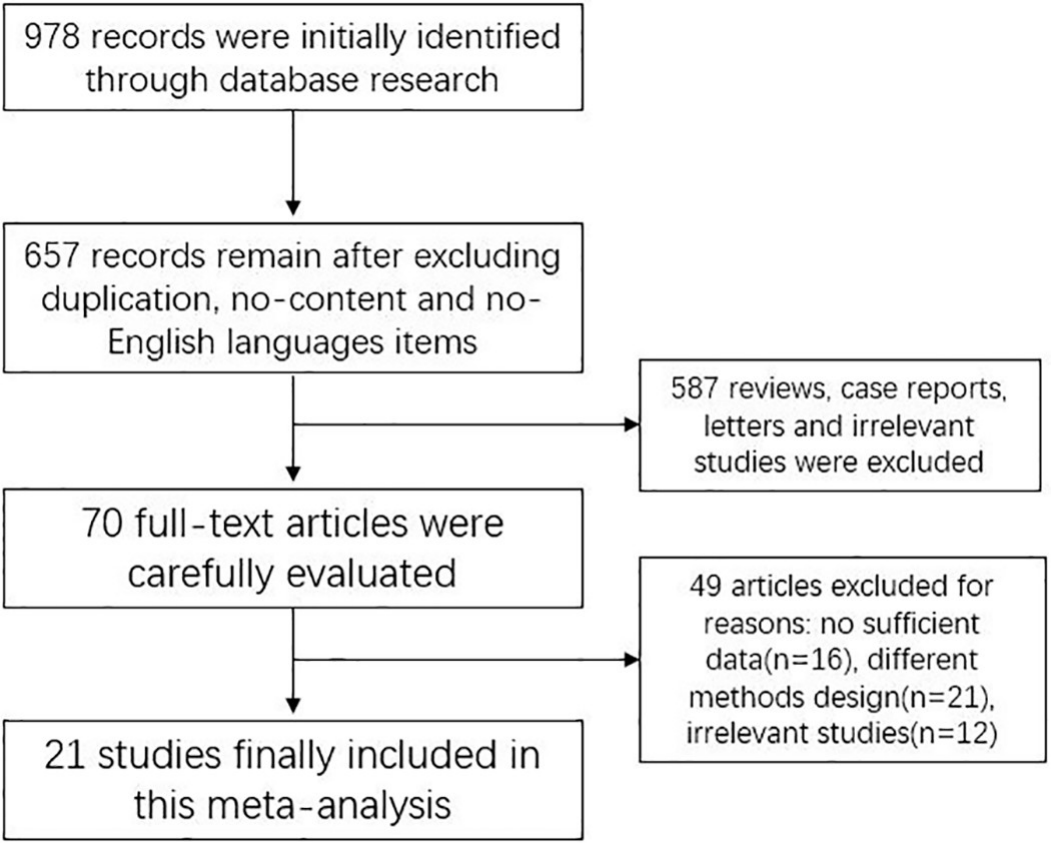
Flow chart of the selection process in this meta-analysis.

After systematically searching the databases, 978 studies were initially retrieved. After identifying items without content and studies published in non-English languages, 321 studies were excluded. Then, the titles and abstracts were carefully scanned, and 587 reviews, case reports, letters and irrelevant studies were excluded. The full texts of the remaining 70 articles were carefully evaluated, and 21 studies that met the inclusion and exclusion criteria were ultimately incorporated in this meta-analysis [10–30]. In total, 6505 patients were included in this meta-analysis, and amputation occurred in 2006 patients who were diagnosed with DFU. The basic characteristics of the included studies are summarized in Table 1. A flow chart of the selection process of the studies included in this meta-analysis is shown in Fig 1.

**Table 1:** Basic characteristics of the included studies.

Author	Year	Study design	Country/Region	Sample size (LEA/total)	Rate %	Quality assessment
Ugwu, E. [11]	2019	prospective	Nigeria	119/336	35.42	7
Sayiner, Z. A. [12]	2019	retrospective	Turkey	143/400	35.75	6
Jeyaraman, K. [13]	2019	retrospective	Australia	263/513	51.27	6
Guo, Z. [14]	2019	retrospective	China	59/475	12.42	6
Kim, S. Y. [15]	2019	retrospective	Korea	28/73	38.36	6
Musa, I. R. [16]	2018	prospective	Saudi Arabia	33/82	40.24	7
Jeong, E. G. [17]	2018	retrospective	Korea	113/192	58.85	6
Ferreira, L. [18]	2018	retrospective	Portugal	48/479	10.02	6
Saleem, S. [19]	2017	prospective	Pakistan	31/107	28.97	7
Jeon, B. J. [20]	2017	retrospective	Korea	67/137	48.91	6
Jiang, Y. [21]	2015	retrospective	China	133/669	19.88	6
Chuan, F. [22]	2015	retrospective	China	62/364	17.03	6
Blumberg, S. N. [23]	2014	retrospective	USA	99/234	42.31	5
Zubair, M. [24]	2012	prospective	India	46/162	28.40	7
Sun, J. H. [25]	2012	retrospective	China	338/789	42.84	6
Li, X. [26]	2011	retrospective	China	112/520	21.54	6
Aydin, K. [27]	2010	retrospective	Turkey	16/74	21.62	6
Yesil, S. [28]	2009	retrospective	Turkey	213/574	37.11	6
Al-Tawfiq, J. A. [29]	2009	prospective	Saudi Arabia	12/62	19.35	6
Mehmood, K. [30]	2008	retrospective	Pakistan	17/116	14.66	6
Gurlek, A. [31]	1998	retrospective	Turkey	54/147	36.73	6

### Sex

**Fig 2:**
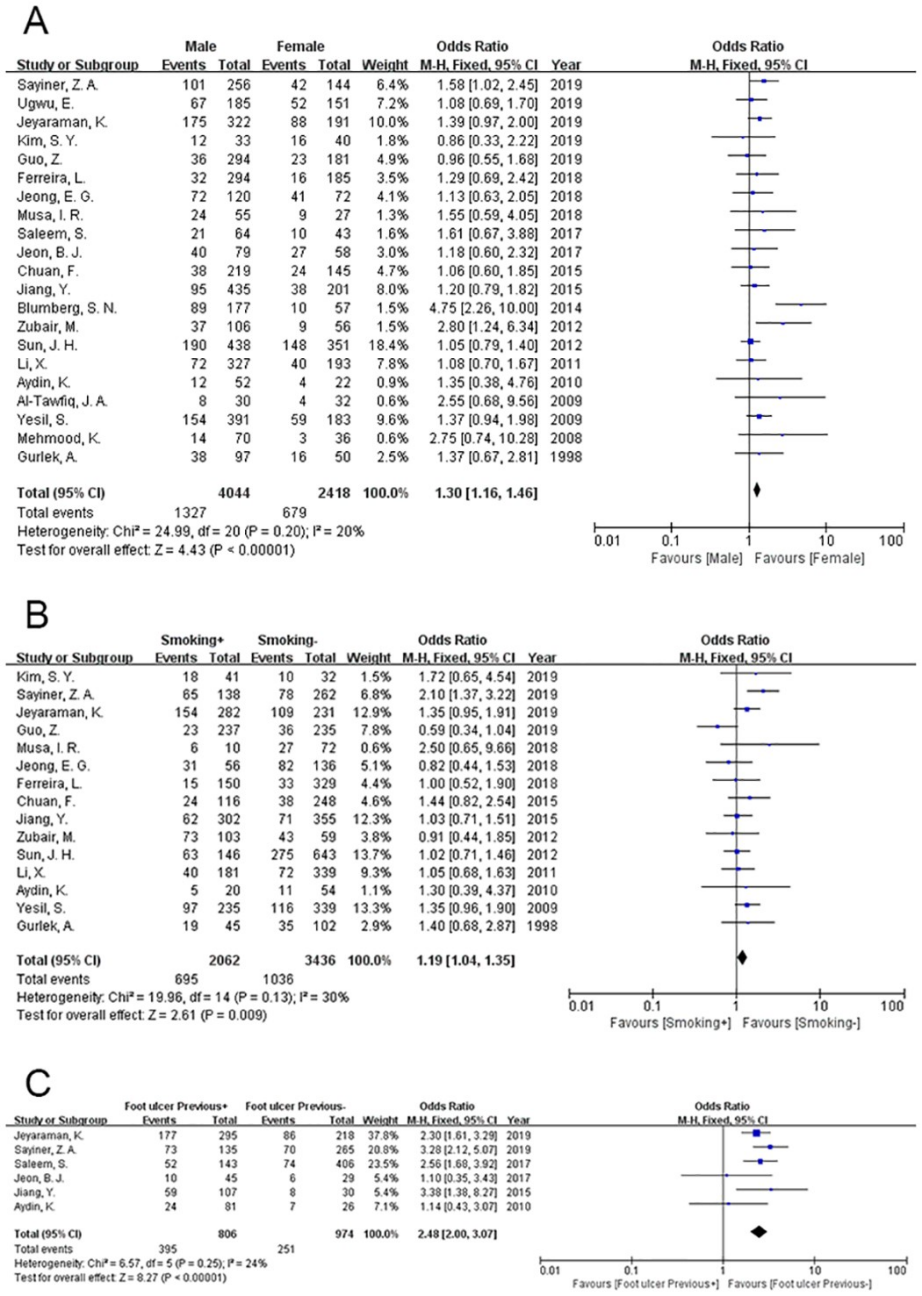
Meta-analysis results of the occurrence of LEA in the two groups. (A) Sex; (B) Smoking history; (C) History of foot ulcers.

The data were analysed using a fixed-effects model (P = 0.20, I^2^ = 20%). The incidence of amputation was 32.81% in the male DFU patients and 28.08% in the female patients. The male patients with DFU had a significantly higher incidence of amputation (OR = 1.30, 95% CI = 1.16~1.46, P<0.00001) (Fig 2A).

### Smoking history

Smoking history is defined as past or present smoking. Many DFU patients have a history of smoking. Therefore, we evaluated the influence of smoking history on the amputation incidence in DFU patients. A fixed-effects model was used because of the lower heterogeneity of the data (P = 0.13, I^2^ = 30%). Five papers were included in this analysis, and the results revealed that a smoking history was among the factors influencing the incidence of amputation in DFU patients (OR = 1.19, 95% CI = 1.04~1.35, P = 0.009) (Fig 2B).

### History of foot ulcers

Patients who previously suffered from foot ulcers were included in this meta-analysis. A fixed-effects model was applied to this data analysis (P = 0.25, I^2^ = 24%), and 6 studies reporting a history of foot ulcers were included. The analysis indicated that DFU patients with a previous history of ulceration are more prone to amputation (OR = 2.48, 95% CI = 2.00~3.07, P<0.00001) (Fig 2C).

### Osteomyelitis

**Fig 3:**
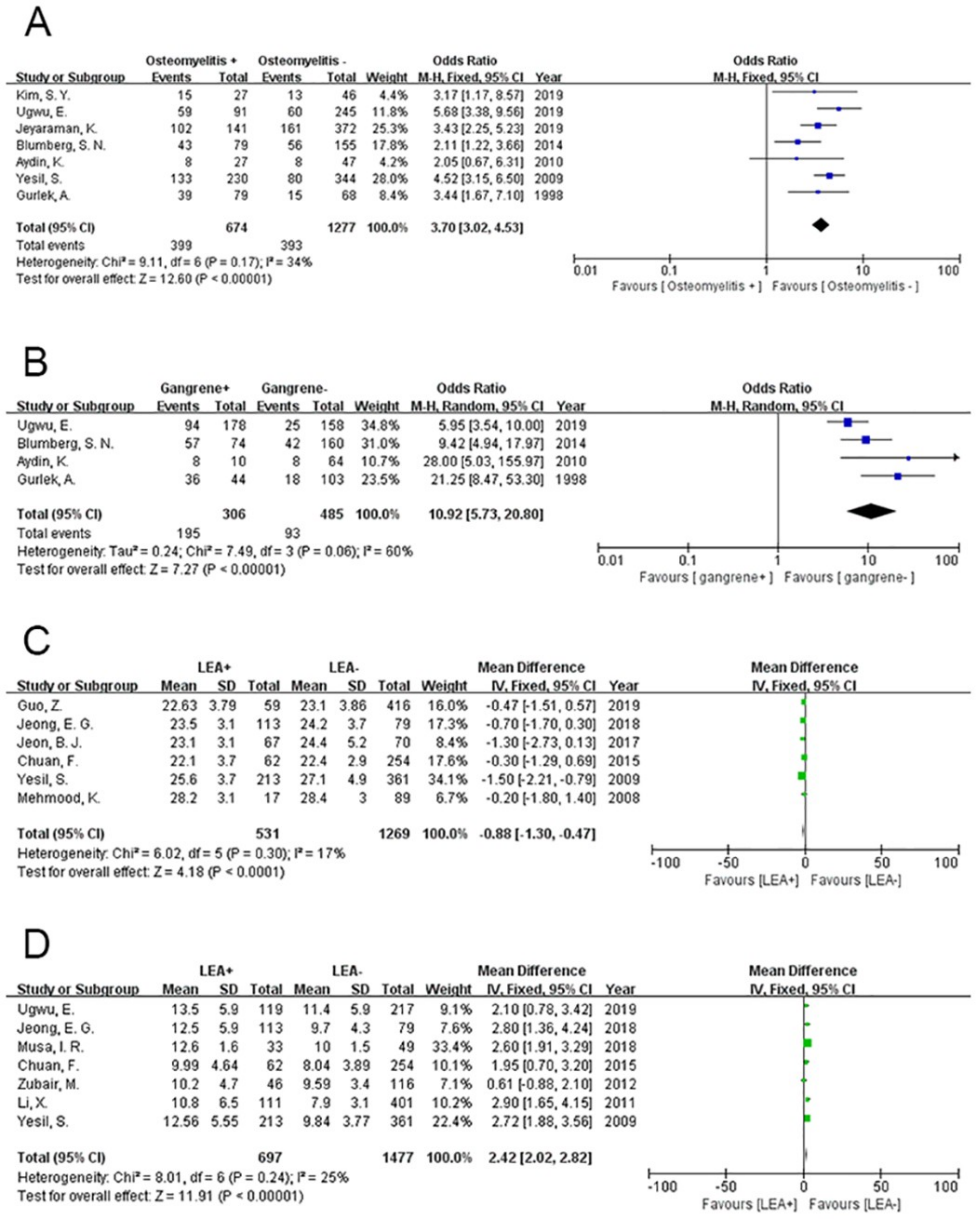
Meta-analysis results of the occurrence of LEA in the two groups. (A) Osteomyelitis; (B) Gangrene; (C) Body mass index; (D) WBC count.

A fixed-effects model was used to analyse the data of the DFU patients with osteomyelitis (P = 0.17, I^2^ = 34%). In total, 7 studies that revealed the effect of osteomyelitis on the incidence of amputation in DFU patients were included in this analysis. The analysis clearly illustrates that osteomyelitis can increase the incidence of amputation caused by DFU (OR = 3.70, 95% CI = 3.02~4.53, P<0.00001) (Fig 3A).

### Gangrene

The data were analysed by a random-effects model to distinguish the relationship between the appearance of gangrene and amputation among DFU patients (P = 0.06, I^2^ = 60%). In total, 4 articles were included, and the results showed that the DFU patients with gangrene had a higher incidence of amputation (OR = 10.90, 95% CI = 5.73~20.8, P<0.00001) (Fig 3B).

### Body mass index

Six studies revealed the relationship between body mass index (BMI) and amputation in DFU patients. We used a fixed-effect model for the data analysis (P = 0.30, I^2^ = 17%) and found that DFU-related amputations were more likely to occur in patients with a lower BMI (MD = -0.88, 95% CI = -1.30~-0.47, P<0.0001) (Fig 3C).

### White blood cell count

White blood cell (WBC, 10^9^ cells/L) count data from 7 papers were analysed by a fixed-effects model (P = 0.24, I^2^ = 25%). The results of the meta-analysis revealed that there was a significant difference between the LEA and no-LEA groups (MD = 2.42, 95% CI = 2.02~2.82, P<0.00001) (Fig 3D).

### Age

**Fig 4:**
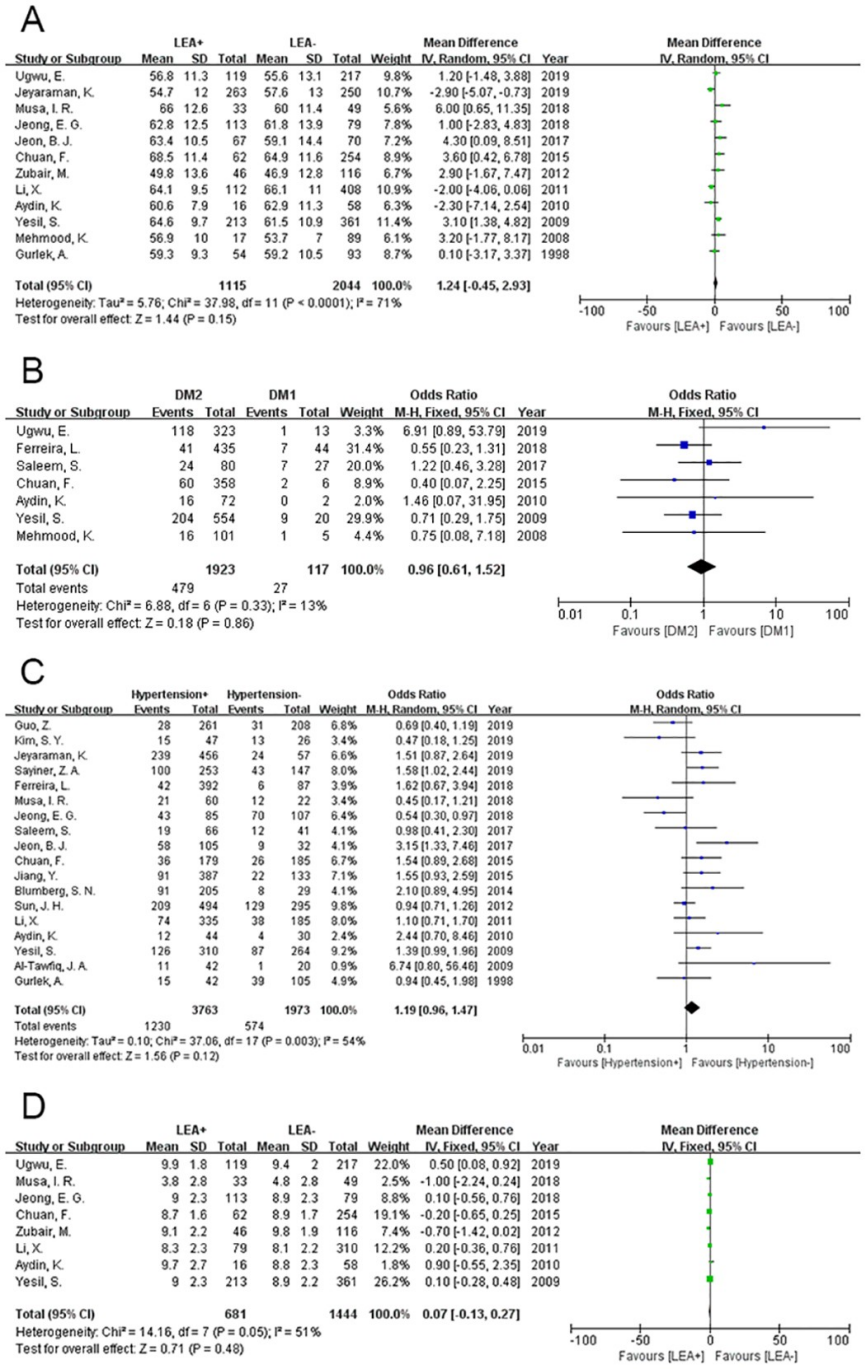
Meta-analysis results of the occurrence of LEA in the two groups. (A) Age; (B) Type of diabetes; (C) Hypertension; (D) HbA1c.

In total, 12 studies analysed the relationship between age and amputation due to DFUs. Since the data were expected to exhibit high heterogeneity, the random-effects model was suitable for the analysis (P<0.0001, I^2^ = 71%). The results showed no clear evidence that age is a factor that changes the incidence of amputation in DFU patients (MD = 1.24, 95% CI = -0.45~2.93, P = 0.15) (Fig 4A).

### Type of diabetes

Both type 1 diabetes mellitus and type 2 diabetes mellitus can cause foot ulcers. We explored whether different types of diabetes mellitus affect the amputation incidence in DFU patients in this meta-analysis. Relevant data from 7 studies were analysed by a fixed-effects model (P = 0.33, I^2^ = 13%), and the results indicated that foot ulcers caused by different types of diabetes did not influence the amputation incidence (OR = 0.96, 95% CI = 0.61~1.52, P = 0.86) (Fig 4B).

### Hypertension

In this meta-analysis, hypertension was defined as systolic blood pressure ≥140 mmHg, diastolic blood pressure ≥90 mmHg, or the use of antihypertensive medication. A random-effects model was used for the data analysis (P = 0.003, I^2^ = 54%). Eighteen relevant studies were included in the analysis, and the incidence of amputation in DFU patients was not associated with hypertension (OR = 1.19, 95% CI = 0.96~1.47, P = 0.12) (Fig 4C).

### HbA1c

Eight articles collected patients’ HbA1c data, and these data were analysed by using a random-effects model (P = 0.05, I^2^ = 51%). The analysis showed that the HbA1c level in patients with DFU does not affect the incidence of amputation (MD = 0.02, 95% CI = -0.28~0.33, P = 0.87) (Fig 4D).

**Fig 5:**
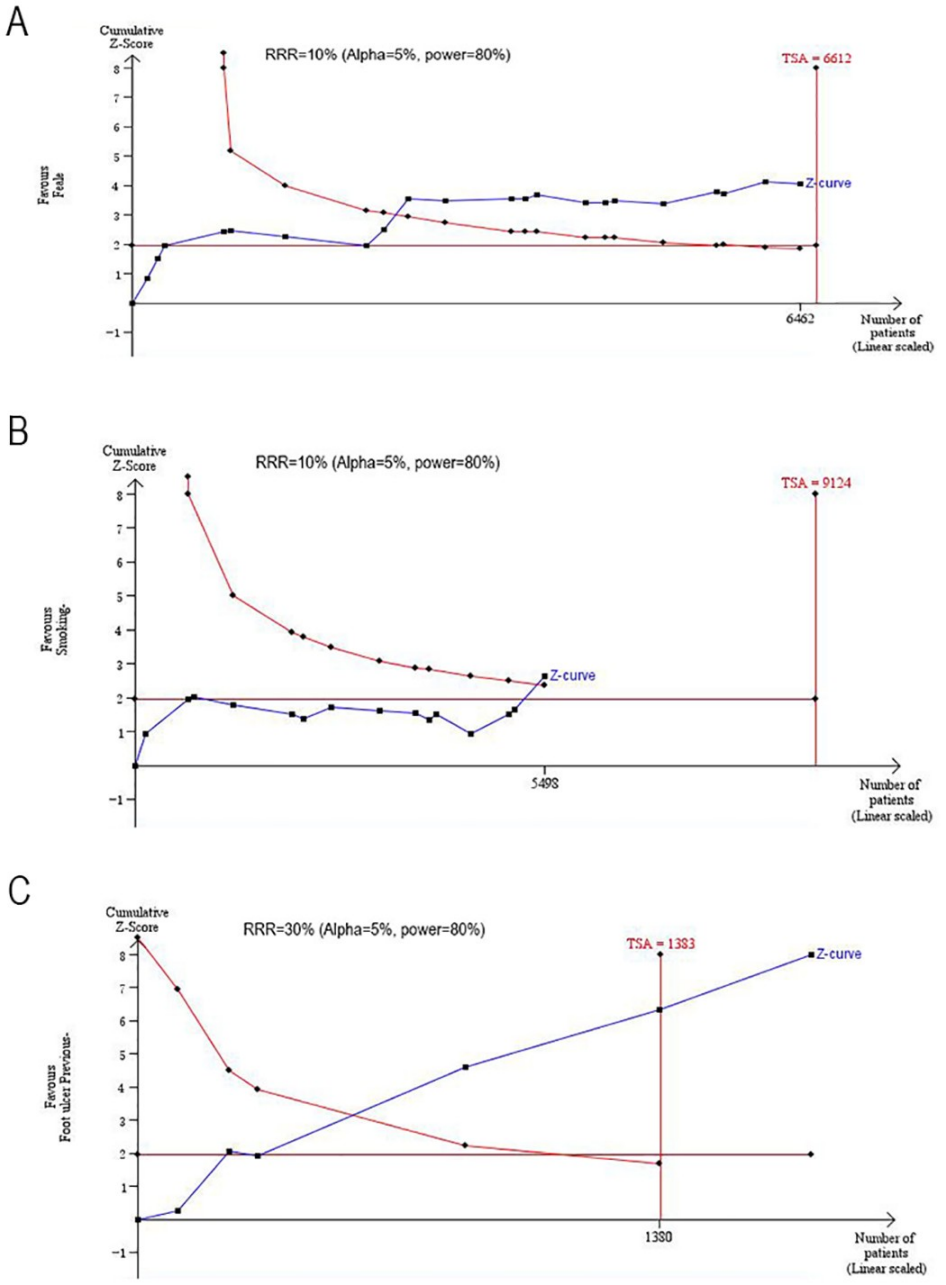
TSA results of the related risk factors. (A) Sex; (B) Smoking history; (C) History of foot ulcers. The information size was calculated based on relative risk reduction (RRR), alpha of 5%, power of 80%.

**Fig 6:**
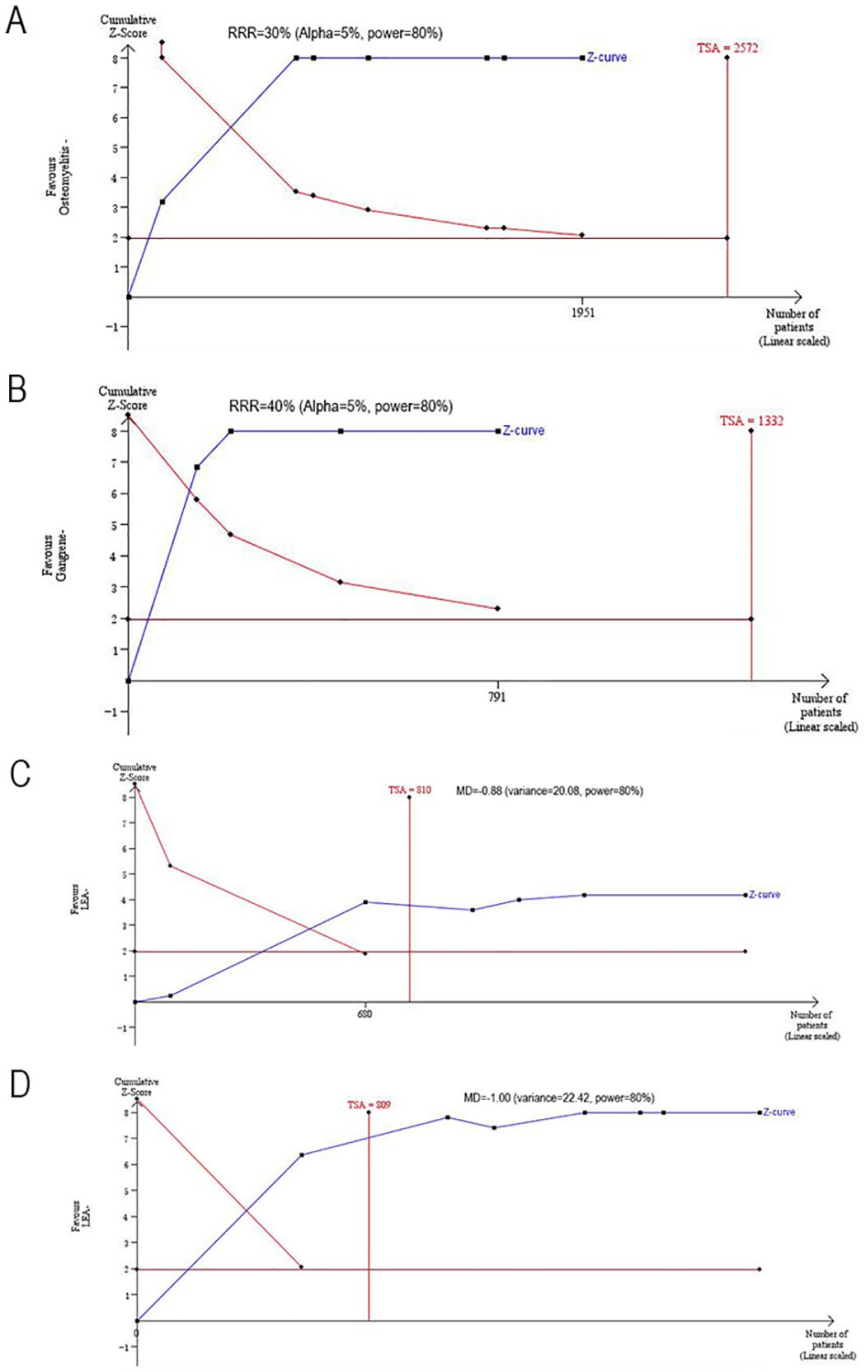
TSA results of the related risk factors. (A) Osteomyelitis; (B) Gangrene; (C) BMI; (D) WBC count. The information size was calculated based on RRR or MD, alpha of 5%, power of 80%.

The pooled outcomes of all items in our meta-analysis are shown in Table 2. We conducted a TSA of the related risk factors, including sex, smoking history, history of foot ulcers, osteomyelitis, gangrene, BMI and WBC count. The results show that the sample size of the above factors is sufficient to obtain significant results, and figures illustrating the TSA are provided in Figs 5 and 6. The horizontal red line represents the boundary at P = 0.05. A vertical horizontal line indicates the required information size. This Z-curve crosses the boundary, indicating that the sample size is adequate.

**Table 2:** Pooled outcomes of all factors.

Risk factors	Studies	Statistical model	OR/MD	95% CI	*P*-value
Sex	21	Fixed-effects	1.30	1.16~1.46	<0.00001
Smoking history	15	Fixed-effects	1.19	1.04~1.35	0.009
Ulcer history	6	Fixed-effects	2.48	2.00~3.07	<0.00001
Osteomyelitis	7	Fixed-effects	3.70	3.02~4.53	<0.00001
Gangrene	4	Random-effects	10.92	5.73~20.80	<0.00001
BMI	6	Fixed-effects	-0.88	-1.30~-0.47	<0.0001
WBC count	7	Fixed-effects	2.42	2.02~2.82	<0.00001
Age	12	Random-effects	1.24	-0.45~2.93	0.15
Type of diabetes	7	Fixed-effects	0.96	0.61~1.52	0.86
Hypertension	18	Random-effects	1.19	0.96~1.47	0.12
HbA1c	8	Fixed-effects	0.07	-0.13~0.27	0.48

## Discussion

LEA is among the most feared complications in diabetic patients. Once the first LEA occurs, up to 50% of patients require another amputation within 3–5 years [31, 32]. Numerous studies have revealed the risk factors for amputation in DFU patients, but there are still differences in some relevant factors. To determine the factors that predict the risk of LEA, we examined data from a cross-section of 21 studies conducted in 10 countries. The data included in our analysis showed very large differences in the incidence of LEA in the literature (ranging from 10.02% to 58.85%) (Table 1), and the total incidence rate was 30.84% (2006/6505). In addition, most studies (13/21) reported amputation rates greater than 25% in DFU patients.

In this meta-analysis, males exhibited a 1.30-fold increased risk of LEA compared to females, but the reason for the difference is unclear. One explanation for the correlation between the male sex and amputation incidence might be the inferior level of foot care among male patients compared to female patients as follows: men do not view their feet as often as women and may visit a physician later than women in the case of any foot problems. Women are usually more motivated to care for their health than men [33]. The literature strongly suggests that men and women differ in their responses to pain as follows: responses to pain are more variable among women than men with increased pain sensitivity and many more painful diseases commonly reported among women [34]. Furthermore, men's roles in society and family may cause them to ignore minor changes in foot lesions. Therefore, men may discover the severity of foot ulcers later than women, which may ultimately affect the outcome of DFU.

It has been widely acknowledged that smoking is a leading risk factor for peripheral arterial disease, cardiovascular disease, and total mortality in the general population [35–37]. However, the direct causal association between tobacco use and foot ulceration or amputation remains controversial [2]. The relevant studies included in this meta-analysis considered only the effects of smoking history on amputation but did not record the detailed statistics of the smoking frequency, amount of smoking, duration of smoking, or types of tobacco. Nevertheless, the results of this study suggest that a history of smoking is a risk factor for LEA in DFU patients; thus, DFU patients may benefit from strict smoking cessation.

A history of ulcers increases the risk of another ulcer in DFU patients [38]. Unsurprisingly, 20% to 58% of patients develop another ulcer within one year of wound healing [39]. Our study found that these recurrent foot ulcer patients ultimately had a 2.23-fold amputation rate higher than patients with first-time foot ulcers. The recurrence of foot ulcers suggests that the factors leading to foot ulcers still exist [40]. Without sustained and effective interventions, these factors may lead to the further progression of ulcers and eventually irreversible limb loss. Therefore, for patients with repeated foot ulcers, we need to identify the relevant factors that may lead to their recurrence and adopt effective measures to avoid the recurrence and progression of ulcers, which may save the patients’ limbs.

BMI is a universal international standard used to measure body weight. To further explore whether obesity affects the outcome of DFU patients, we compared and analysed the differences in BMI between amputated and nonamputated DFU patients. We found that a lower BMI resulted in a higher incidence of amputation in patients with DFU. Diabetic foot, especially those with infection, is a type of consumptive disease. Patients with a higher BMI may have a better nutritional status and, hence, more strength to cope with a severe illness [41]. The apparent protective effect of a higher BMI on the foot ulcer risk may be an instance of ‘the obesity paradox’, a phenomenon of better health outcomes associated with an elevated body weight [42].

The acute phase response in DFUs mostly depends on limb ischaemia, the severity of infection and the presence of osteomyelitis. Previous studies have found that leukocytosis is a poor indicator of acute osteomyelitis, and a WBC count>12.0×10^9^ cells/L is related to an increased risk of amputation [43]. Based on the data included in this study, the average WBC count in the DFU patients with amputation is close to this value (11.75×10^9^ cells/L), while the WBC count in the patients who did not have amputations was only 8.83×10^9^ cells/L. The difference between these two groups suggests that foot infection and the acute phase response play an important role in the progression of DFUs to amputation. Our meta-analysis also reveals that once osteomyelitis or even gangrene occurs, the risk of amputation in diabetic foot patients rapidly increases. Therefore, for DFU patients complicated with infection, especially those who progress to osteomyelitis and gangrene, adequate debridement, high-quality wound care and effective anti-infection may reduce the risk of amputation.

Diabetes has traditionally been subdivided into type 1 and type 2 diabetes. All included studies used this classification to analyse whether different types of diabetes affect amputation outcomes in DFU patients. Although there are some differences between these two types of diabetes in terms of age, aetiology and symptoms [44, 45], neither type 1 nor type 2 diabetes has been considered a risk factor for LEA in DFU. Over the past few decades, the notion of diabetes has widened with the deepening understanding of this lesion. This classification method has been considered a gross oversimplification that poorly describes the true range of diabetes [46]. However, due to the lack of relevant data, whether more detailed classification methods are related to amputation in DFU patients remains unclear.

Hypertension and diabetes mellitus are common diseases. Hypertension is twice as common in diabetics than nondiabetics [47]. In the EUROASPIRE IV survey, only 54% of diabetic patients had a blood pressure below 140/90 mmHg [48]. In this meta-analysis, approximately 65.60% of the DFU patients had concurrent hypertension (3763/5736). These data suggest that hypertension may be an important factor affecting the prognosis of DFU patients. However, after the analysis, we found that hypertension did not increase the risk of amputation in DFU patients. Nonetheless, it is still necessary for DFU patients to control their blood pressure as hypertension may lead to adverse cardiovascular and cerebrovascular events.

As a glycaemic control parameter, HbA1c showed no difference between the LEA group and the non-LEA group in this meta-analysis. A possible explanation for the current finding is that there was no significant difference in the baseline HbA1c between the amputees and nonamputees [25]. HbA1c can reflect the glycaemic control level of diabetics over the last several months, while DFUs are a long-term pathological process. After receiving recent intensive treatment, the detrimental effect of pre-existing poor diabetic control at the entry point of the study could be offset by the aggressive treatment [25]. However, previous studies have found that the benefits of glycaemic control are not evident in macrovascular outcomes [49]. Therefore, HbA1c may not be a predictor of amputation in DFU patients. Nevertheless, since the included studies were retrospective and lacked continuous data, our results cannot negate the role of glycaemic control in the prognosis of DFU patients. More prospective studies are required to confirm whether long-term glycaemic control affects the occurrence of amputation.

### Limitations

There are several limitations to this study that must be considered when interpreting the results. Although the risk factors of major and minor amputations may differ, we failed to determine the risk factors of major and minor amputations separately due to the limited data in the included studies. In total, 21 papers are included in our meta-analysis, but most papers represent retrospective studies. Eight of the 21 included studies were from East Asia, which may have caused bias. Different hospitals in different regions have different diagnostic, therapeutic capabilities and medication use, which may affect the prognosis of patients. Several included studies contained significantly more patients than other studies, and these trials may lead to bias in assessing the outcome of our study. The differences in the study populations and aims of the included studies might lead to selection bias. Although some studies reported that some diabetic complications are related to DFU or amputation, due to the inconsistencies in the diagnostic criteria, we failed to analyse some other possible risk factors, such as diabetic nephropathy, diabetic retinopathy, diabetic peripheral neuropathy, coronary artery disease, peripheral arterial disease, and history of stroke. Diabetic foot is a long-term pathological process, and the included retrospective studies cannot provide longer follow-up data, which may have an impact on the results of the study.

## Conclusion

In conclusion, DFU is among the most common, serious and costly complications of diabetes. Our meta-analysis identified the following significant risk factors for amputation in DFU patients: the male sex, a smoking history, a history of foot ulcers, osteomyelitis, gangrene, a lower BMI, and a higher WBC count. Once gangrene occurs, the risk of diabetic foot-related amputation rapidly increases. We also found that age, hypertension, type of diabetes, and the HbA1c level were not related to the occurrence of amputation in DFU patients. Although some risk factors are difficult to reverse, knowledge of these factors and their influence on amputation outcomes is critical for allowing multidisciplinary teams to develop management and treatment protocols for patients with DFU.

## Supporting information

S1 Fig Funnel plots. (TIF)

S1 Table PRISMA checklist. (DOC)

S2 Table Raw data. (XLSX)
