# Supplementary figures and images for: Risk factors for lower extremity amputation in patients with diabetic foot ulcers: A meta-analysis
Source: PLoS One. 2020 Sep 16;15(9):e0239236. doi: 10.1371/journal.pone.0239236 (PMC7494323; doi:10.1371/journal.pone.0239236)

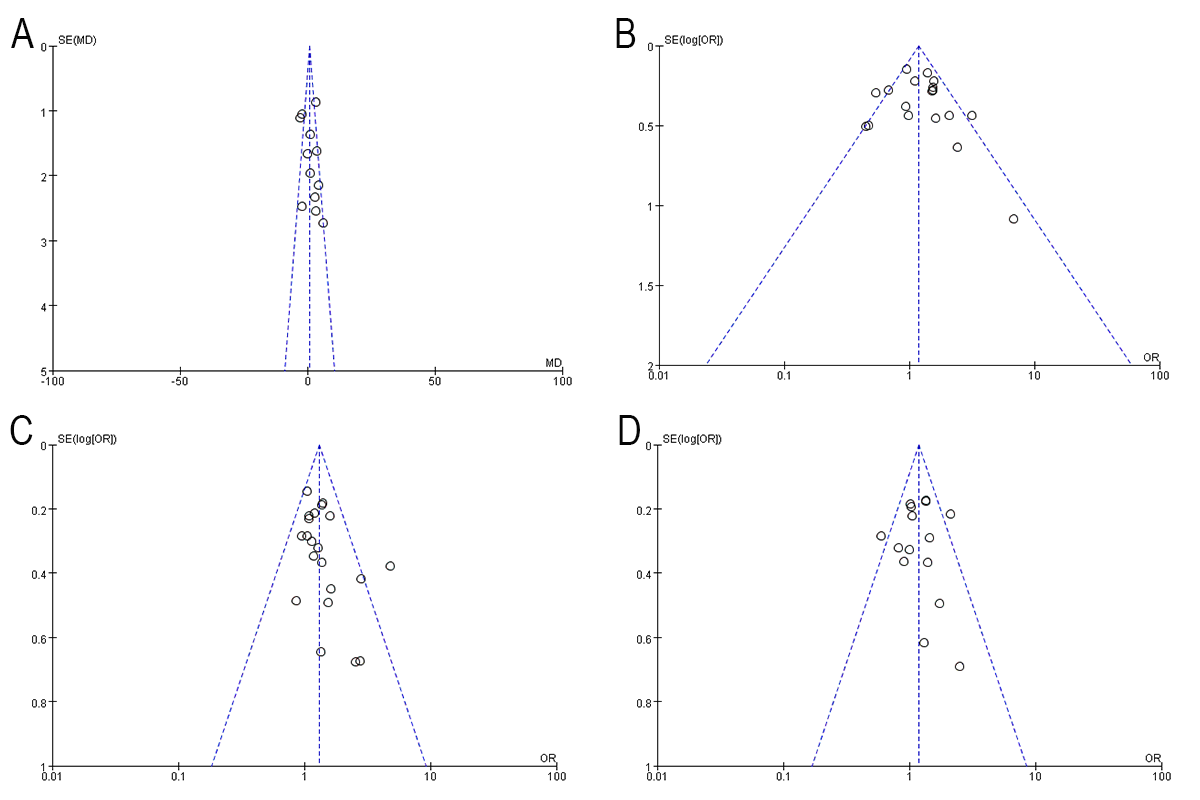

Supplement: S1 Fig — (TIF) [file pone.0239236.s001.tif]
